# A retrospective radiographic evaluation of the anterior loop of the mental nerve: Comparison between panoramic radiography and cone beam computerized tomography

**DOI:** 10.4317/medoral.20026

**Published:** 2014-12-31

**Authors:** Aleksandar Vujanovic-Eskenazi, Jesus-Manuel Valero-James, María-Angeles Sánchez-Garcés, Cosme Gay-Escoda

**Affiliations:** 1 BSc, BDS, MFDS RCS Ed, Fellow in Oral Surgery and Implant Dentistry, School of Dentistry, University of Barcelona, Spain; 2 DDS, Fellow in Oral Surgery and Implant Dentistry, School of Dentistry, University of Barcelona, Spain; 3 MD, DDS, PhD, EBOS, Assistant Professor of Oral and Maxillofacial Surgery. Master’s Degree Program in Oral Surgery and Implantology, School of Dentistry, University of Barcelona, Barcelona, Spain. Researcher of the IDIBELL Institute Barcelona, Spain; 4 MD, DDS, PhD, MS, EBOS, Chairman and Professor and of Oral and Maxillofacial Surgery Department. Director of Master’s Degree Program in Oral Surgery and Implantology, School of Dentistry, University of Barcelona. Coordinator/Researcher of the IDIBELL Institute, Barcelona, Spain. Head of the Department of Oral and Maxillofacial Surgery of the Teknon Medical Center, Barcelona, Spain

## Abstract

Objectives: To compare the prevalence and the length of mental loop, measured with panoramic radiography (PR) and cone beam computerized tomography (CBCT). Material and Methods: PG and CBCT images where analyzed by a single calibrated examiner to determine the presence and the position of the mental foramen (MF), its distance to the lower mandible border, the anterior length of the mental loop (ML) and the bone quality in 82 PR and 82 CBCT. Results: ML was identified in 36.6 % of PR and 48.8 % of CBCT. PR showed a magnification of 1.87 when compared to CBCT. The mean of anterior extension of the inferior alveolar nerve and the distance to the inferior border of the mandible was higher for PR (2.8 mm, sd 0.91 mm on the PR , range 1.5 to 4.7 mm and 1.59, sd 0.9 on the CBCT ,range 0.4 to 4.0 mm) Conclusions: There is a magnification in PR images with respect to those of CBCT. The differences between CBCT and PR with regards to the identification and length of the ML are not statistically significant. Identification and accuracy measurements of ML did not depend on the bone quality. Considering that two dimensional imaging provides less accurate and reliable information regarding the anterior loop, a CBCT scan could be recommended when planning implant placement in the anterior region.

** Key words:**Mental loop, mental nerve, mental canal, preoperative implant planning, panoramic tomography, cone beam computerized tomography.

## Introduction

The final portion of the inferior alveolar nerve sometimes passes bellow the inferior border and the anterior wall of the mental foramen and, after giving off a small incisive branch, it curves back to enter the foramen and emerge to the soft tissues becoming the mental nerve. This anatomical feature is also known as ‘’anterior loop’’ of the inferior alveolar nerve.

Correct placement of dental implants in the mental region may be limited by this anatomic structure and its violation can lead to a neurosensory disturbance in the area of the lower lip.

To avoid such a trauma a 5mm safe distance from the foramen has been proposed for chin bone harvesting (1) and implant placement.

Usually the panoramic radiography (PR), together with the clinical examination, has been used as the only preoperative diagnostic tool in treatment planning of implant placement in interforaminal region. Even though the magnification shown by PR is small in the anterior region, the distortion and the magnification of the anatomical structures is often present, and results in either over- or underestimation of the real size (2,3).

Other diagnostic techniques, such as intraoral periapical radiographs and CT do not allow exact measurements nevertheless, CT provides more reliable data and can be used to obtain detailed information using a three-dimensional analysis (4).

A new possibility for improved diagnostic imaging is the Cone Bean Computer Tomography (CBCT). The images obtained when performing CBCT are similar to those found on CT. CBCT is an advanced digital imaging technique that allows to generate multi planar slices of a region of interest and is capable to reconstruct a 3D image using a cone-shaped rotating x-ray beam via a series of mathematical algorithms (5). Currently it is being increasingly used based on the good relation cost-effectiveness, shorter time of acquisition, higher power of resolution level and 1/15 less radiation exposure compared to CT (6).

Studies focusing on checking the accuracy of the measurements taken by CT and CBCT on patients versus those taken in cadavers, CBCT showed the lowest magnification (7) and there is no significant difference in images magnification depending on tooth location either or vertical and mesiodistal direction (8). Also it is possible to determine a relative quality of bone, select a smaller field of view and have the possibility to manufacture a CBCT-surgical guide with the aid of a software package, all as an in-office modality. There is a Consensus Report about the use of CBCT in implant dentistry with the intent of providing a scientifically based guidance to clinicians regarding its use as an adjunct to traditional imaging modalities (5).

In a study by Parnia *et al*. (9) after evaluating 96 CBCT, the mandibular foramen could be detected in 100% of cases, anterior loop in 84%, incisive canal 83% and lingual foramen in 49% of the images. The mean size of the anterior loop was 3.54mm± 1.41, and the authors have concluded that due to great variability in the length of anterior loop , it is not safe to recommend any definite distance mesially from the mental foramen when a dental implant has to be placed. This length data is similar to that found on PRs in our previous study (3.6mm± 0.78) (10) but differs considerably from that registered in the study by Apostolakis and Brown (0.89mm±1.17) (11).

The aim of the present study is to describe the size of the mental neurovascular bundle and to compare the measurements obtained from PR with those obtained from CBCT. Our hypothesis is that CBCT offers lower magnification than PR and this will ideally be represented by bigger mean value of mental loop measurements.

The study was carried out by an independent experienced examiner.

(we have delayed a sentence containing we are in agreement with...)

## Material and Methods

A descriptive, retrospective study was conducted including 164 radiographic exams (82 PRs and 82 CBCTs) taken from 82 partially or totally edentulous healthy patients that have consecutively attended our hospital for dental treatment requiring tomographic examination in the bilateral mandible for various clinical indications, and have both PR and CBCT taken between January 2012 and July 2012, with a mean age of 56. 56 years (range 18 to 80), 26 males (31.7%) and 56 females (68.3%).

No exclusions were made due to age and gender. All PR were obtained using the same radiographic equipment (Planmeca ProMax, Helsinki, Finland) as well as for CBCT (Kodak 9000 3D, Carestream Health, Inc., New York, USA). The study was carried out by one observer who has also recorded the bone quality, as described by Lekholm and Zarb (type 1 to 4), depending on the thickness of the cortical bone and density of the bone marrow on a parasagittal plane of each CBCT scan.

-Measurements: All radiographs were measured by the second of the authors who was a fellow in Oral Surgery and Implant Dentistry with some experience in CBCT, PR interpretation and implant dentistry. Before the observation period, the guidelines were discussed and the observer was calibrated in both radiographic systems to recognize the existence and the length of the mental loop. For the development of our guidelines we have undertaken a small pilot study were the length of the anterior loop was measured on a vertical cross section using the capabilities of our software. Ten cases (20 sides) were examined with this method and reexamined two weeks later to evaluate the reproducibility of the recordings.

**Figure 1:**
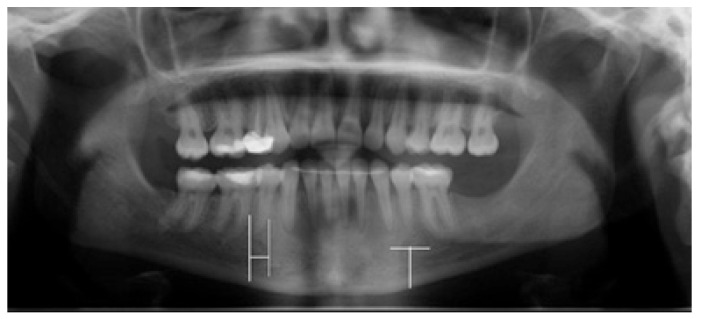
Panoramic radiography. On the right we observe the technique used for the measurement of the mental loop. On the left side we observe the technique used for the measurement of the distance between the mental foramen and the lower border of the mandible.

**Figure 2:**
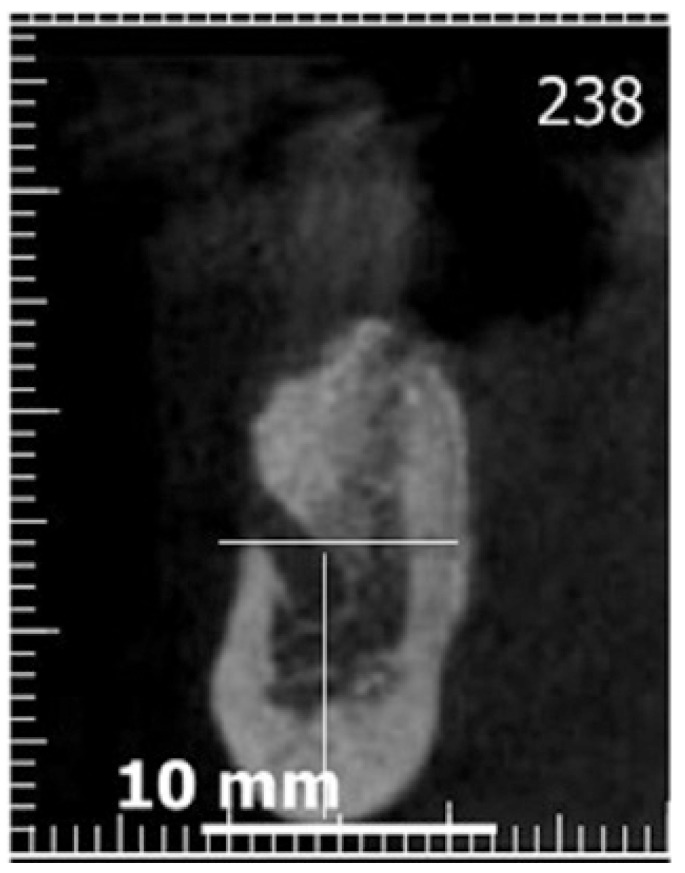
Cross-sectional cut on cone beam computed tomography(CBCT). We observe the technique used for the measurement of the distance between the mental foramen and the lower border of the mandible.

The measurements were taken on the vertical cross section cuts on CBCT and on PR, recording the distance from the lower border of the mandible to the lower point of the mental foramen using a measurement tool provided by each software, selecting on the CBCT the slice showing the lowest level point (Figs. 1,2). A second measurement was done in order to calculate the anterior extension of the mental loop respect to the most mesial point of the mental foramen on PRs, following the guidelines stated by Kuzmanovic *et al*. (2) (Fig. 1), when the mental path described a well identified loop.

**Figure 3:**
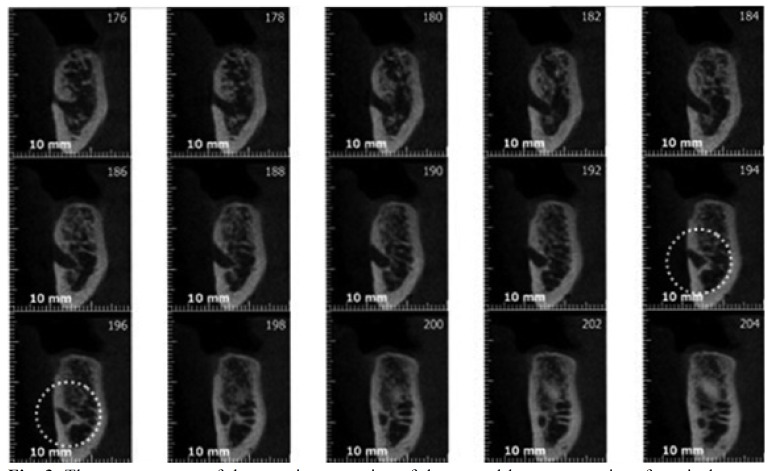
The measurement of the anterior extension of the mental loop on a series of vertical cross-sectional cuts (0.4mm voxel size).

The measurements of the anterior extension performed on CBCT, were done by counting the number of the consecutive contiguous vertical cross sections between the anterior border of the mental foramen and the anterior border of the loop (Fig. 3). This number was multiplied by the thickness of the slices (0.4 mm voxel size).

Furthermore we also measured the distance from the roof of the dental canal to the upper point of the mental foramen in a cross-sectional slice.

Measurement of the mental loop was repeated 2 weeks later to evaluate the intra-observer reproducibility of the recordings.

-Statistical analysis: The statistical analysis was performed with SPSS software version 18 (Chicago, USA License of the University of Barcelona). A descriptive and comparative analysis was performed. Chi- squared tests were used to examine the association between categorical variables such as visibility of the mental loop.

Student´s t- test for paired samples was used to compare the mean length of the mental loop in PR and CBCT.

The intra-class correlation coefficient (ICC) has been calculated for anterior extension of the ML and its distance to the inferior border of the mandible, comparing CBCT and PR.

The prevalence of the anterior loop was calculated and anterior loop length chart was produced.

The results were considered significant where *P* ≤ 0.05.

## Results

The results of Kappa statistics showed 0.42 (*P*= .03) and 0.49 (*P*= .02) reproducibility for panoramic radiography and CBCT, respectively.

The most frequent bone type observed in the interforaminal region was type 3 (53.7%, n=44), followed by type 2 (40.2%, n=33) and type 4 (6.1%, n=5) ( Table 1). No statistical differences were found using Chi- squared test between bone type and the possibility of identifying the mental bundle neither for PR nor for CBCT.

**Table 1:**
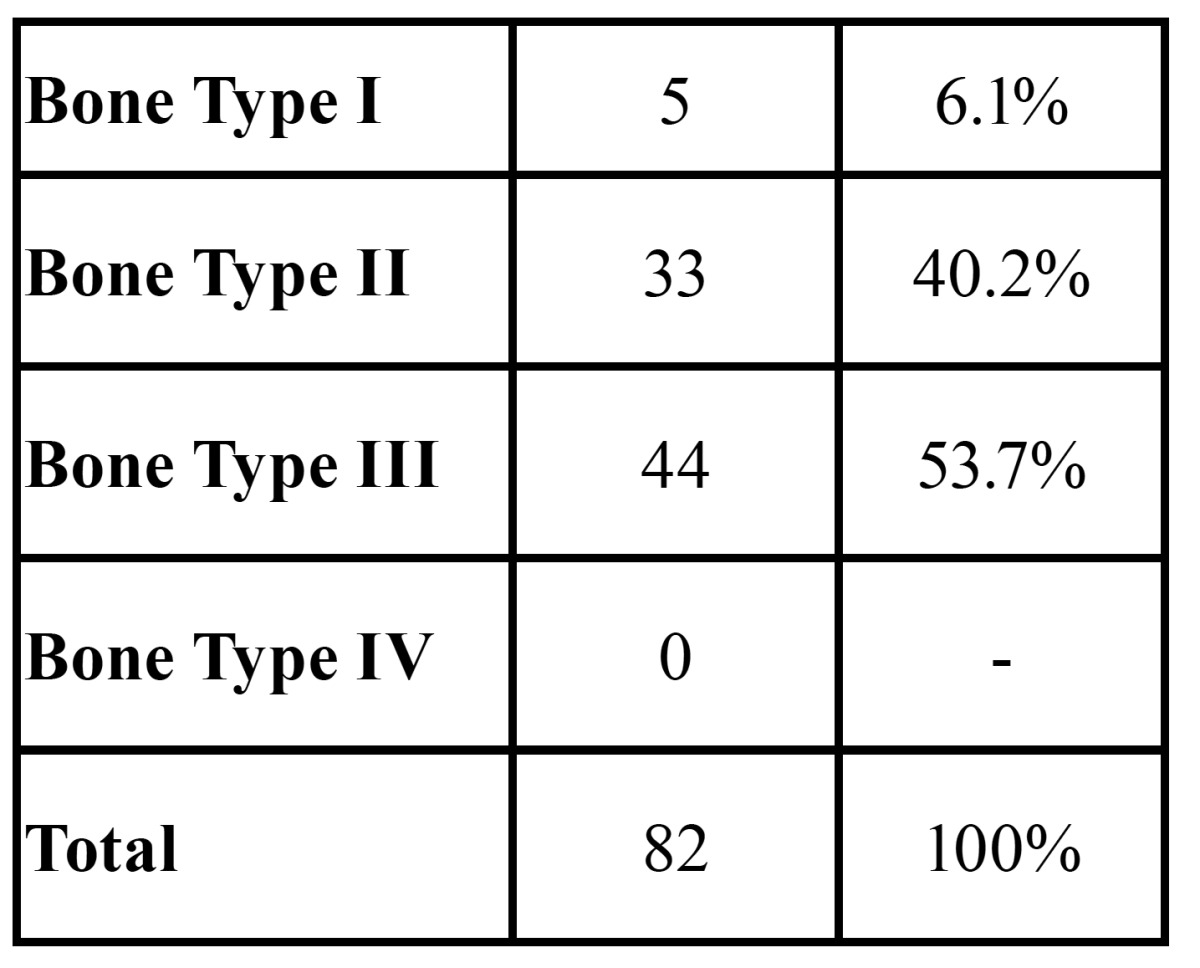
Bone type observed on CBCT as described by Lekholm and Zarb (12).

The mental loop was visible in 36.6% of PR examined as compared to 48.8% of CBCT scans, whereas the mental loop was visible only in 24.4% of patients in both PR and CBCT ( Table 2).

**Table 2:**
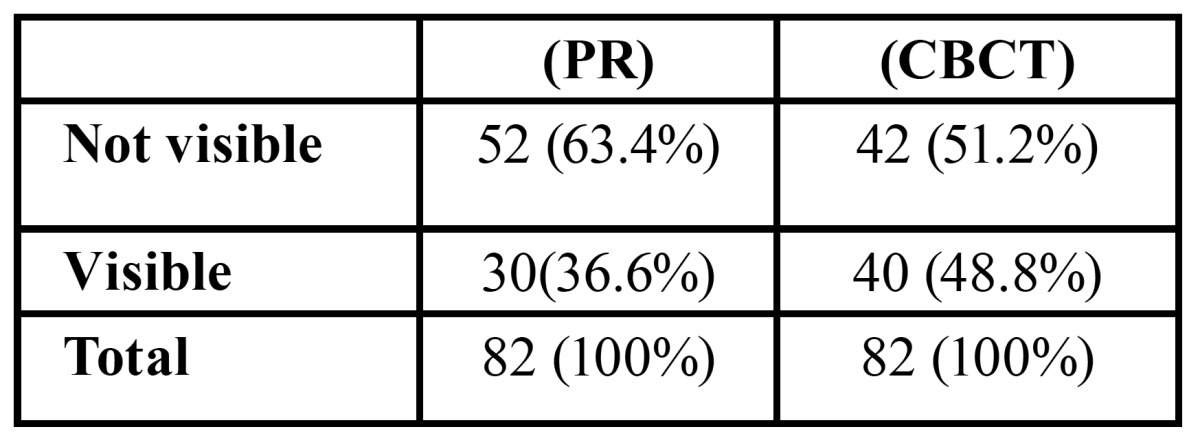
Mental loop visibility on Panoramic Radiograph (PR) and Cone Beam Computed Tomograpfy ( CBCT).

The bone quality did not produce any statistically significant differences for the identification of the ML.

The mean length of the ML was 2.8 mm, sd 0.91 mm on the PR (range 1.5- 4.7 mm) and 1.59, sd 0.9 on the CBCT parasagittal cuts (range 0.4-4.0 mm) ( Table 3).

**Table 3:**
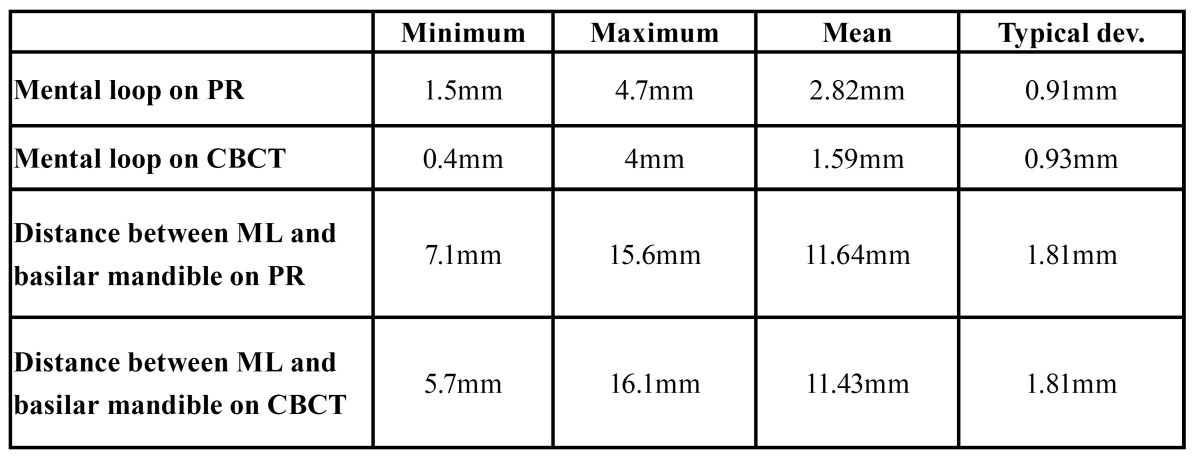
Mean values with typical deviation of the mental loop (ML) and the distance of the mental foramen (MF) and the lower border of the mandible on panoramic radiographies (PR) and cone beam computed tomography (CBCT).

The mean value for magnification of PR in comparison to CBCT scans is 1.87, sd 0.64 for those patients that have had a visible bundle in both PR and CBCT (24.4%).

The mean distance from the lower point of the mental foramen to the lower border of the mandible was 11.6 mm for PRs and 11.4 mm for CBCT.

The mean distance from the roof of the dental canal to the upper point of the mental foramen was 2.17 mm for CBCT.

There is a strong association between the visibility of the mental loop on PRs and CBCTs (p= 0.0014), which set against the nil hypothesis that the bundles on PRs and CBCTs are independent. In other words, there is a tendency for these to either be visible or not on both imaging techniques.

In relation to ICC we have found mayor agreement in the measurement of the distance from the inferior border of the mental loop to the lower border of the mandible (0.587) than the anterior extension of the mental loop (0.225).

## Discussion

To avoid neurological trauma during implant surgery in the interforaminal region, radiographic examination is compulsory. Dysestesia of the lower lip due to mental nerve injury is one of the most serious complications of mandibular implant surgery (12). The risk of sensory disturbance after implant placement in the mental region is a complication that occurs in 7% of the cases when the implants are inserted within 3 mm of the safety margin to the anterior border of the foramen (13) whereas, the incidence of disturbed sensibility after mandibular surgery drops down to 1% when this distance is greater, as it happens when placing 2 implants in the anterior mandible (14).

The panoramic radiography has been used for a long time as the only diagnostic exam in implant dentistry; however in some cases it is not possible to identify the mental foramen or the extension of the anterior loop accurately. These often offer poor resolution and size distortions can be severe depending on the region.

Vertical measurements in PR are less reproducible than horizontal (15) and the image varies between panoramic units (3,16).

The position of the head, the shape of the dental arch, the tooth location, the use and location of a bite block and the type of panoramic equipment could be some factors causing image distortion (16-18). Furthermore panoramic radiographs do not provide accurate information regarding bone quality and quantity available.

In our study all of PR were obtained with the same machine and the position of the head was standardized in a central location using a chin cup.

No corrections were made in respect to the variations of the magnification ratio according to the mandibular area, because all measurements were located in the lower premolar region and in this area distortions are reported as the lowest when compared among other teeth positions (1.152 ± 0.050) (3).

Different studies have shown that panoramic radiographs do not accurately identify the incidence or the extent of the mental loop (2,16), and there is a significant difference in our study between PR and CBCT.

In almost half of our CBCT cases (48.8%) an anterior loop could be identified radio graphically, similar to the results found by Apostolakis and Brown (48%) (11), while anterior looping of the mental nerve was observed in 36.6% of PR images. With regards to the mandibular incisive canal, PR fails to identify this landmark and to verify its existence for preoperative planning purposes.

Considering that CBCT has been used in a limited number of studies for measurements of the anterior loop, this study is useful in demonstrating the value of CBCT in implant dentistry.

The CBCT resembles medical CT on the type of images obtained and in the capability of the software. It has been shown that magnification of CBCT in linear measurements does not occur and that the images are more accurate than those obtained with medical CT (5).

Another important advantage of CBCT is its reduced radiation dose. Although a significant variation in effective dose exist among CBCT machines; however when compared to medical CT, CBCT can be recommended as a dose reducing technique for dental implant application. The effective dose from CBCT examination ranges from 13 µSv to 479 µSv, depending on the available machine. The one used in our study has the range of 14 to 40 µSv. To compare, the effective dose from one PR is approximately 10 to 14 µSv while for a maxillomandibular medical CT ranges from 474 to 1160 µSv (5).

Widely different values are found in the literature for the frequency of anterior loop; in CT studies, the range is 7% (19) to 83% (20); in CBCT studies, values vary between 48 % (11) and 85.2 % (21), coinciding with the results found in our study.

The mean length of the anterior loop was 1.59 mm whilst various studies involving CBCT report mean length ranging from 0.89 mm (9) to 3.54 mm (11). Our longest anterior loop was 4 mm, while the longest loop in the literature is reported by Neiva *et al*. (22) being 11 mm.

In 51.2% of all evaluated cases the mental loop was not visible on CBCT, whereas in 95% of all the cases where the loop did exist, it did not measure longer than 3.6 mm.

These findings are in agreement with Filo *et al*. (23) and Apostolakis *et al*. (11), who reported 95.81% and 95% of the cases measuring up to 3 mm and the longest loop identified of 5.6 and 5.7 mm, respectively. However, despite the fact that the majority of anterior loops extend up to three millimeters, Wismeijer *et al*. (13), found 7% of sensory dysfunction in the lower lip even when a safety margin of 3 mm was used.

Consequently, the safe distance, without the use of CBCT or CT probably would be 6 mm (11), which reduces considerably the interforaminal space for implant placement.

Furthermore, from the prosthetic point of view, the most distal implant should be placed as close as possible to the foramen in order to extend the prosthesis as far as possible and with a cantilever biomechanically favorable.

CBCT is also useful in quantifying and qualifying the inter mental bone and its morphology but not with the same efficiency to locate and measure the dimension of the mental foramen or its anterior loop because it can be easily confused with the incisive canal. Some authors consider that the measurement of the ML is only possible when the mental canal does not show continuity with the mandibular canal in radiographic images (2,16,17), (type I according to Youse & Brooks (17,18), despite all, there is no doubt that measurements on CBCT are more reliable (24,25). In 48.8 % of patients examined in this study the mental loop could be identified on parasagittal cuts of CBCT, this is in disagreement with Kaya *et al*. (16) according to whom ML is more difficult to observe on cross-sectional cuts than on panoramic reconstructions.

As mentioned previously, ML was identified on 36.6 % of the PRs and 48.8% of the CBCTs ( Table 2) and bone quality did not produce any statistically significant differences for the identification of the ML as opposed to Anderson *et al*. (25) and Kaya *et al*. (16) who believe that a possible explanation to the variability in the prevalence of the ML shown on radiological examinations may refer to the low contrast of the poor bone quality cases. In our opinion identification of the ML on PRs is specially related to the degree of cortication of the mental canal wall as also occurs with the identification of the incisive canals.

Extremely different results have been obtained by several authors in terms of identification and measurement of the ML ( Table 4) and this phenomenon also can be explained due to the high anatomical variability. There is a big difference in the visualization of the ML between cadaveric samples (14.3% (26)- 96% (27) and radiographic measurements (7% (19)- 85.2% (21) and the average length of the anterior loop based on direct measurements in cadaveric samples ranged from 0.1 mm and 6.95 mm (2,26-28).

**Table 4:**
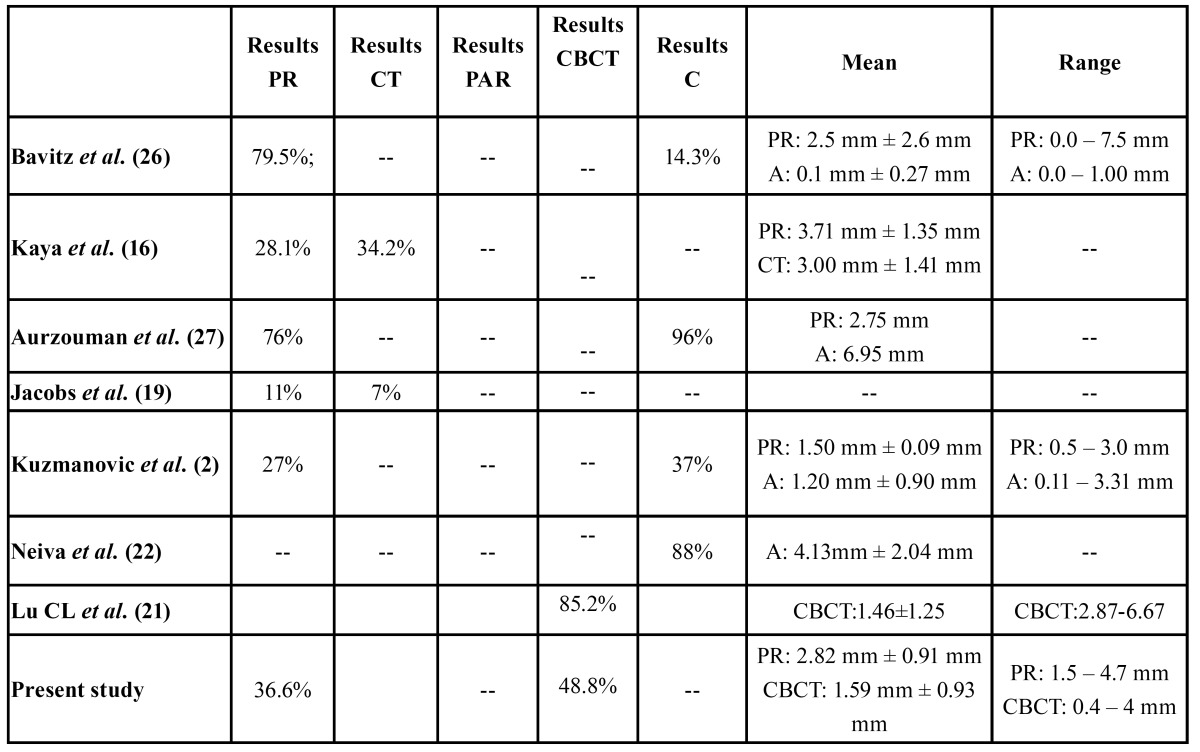
Prevalence and mean values with atypical deviation (A) and range of the mental loop on the panoramic radiography (PR) and the computed radiography (CT) and cone beam computed tomography (CBCT), periapical radiography (PAR) and cadaveric samples (C).Summary of the results found in the literature.

In agreement with Parmia *et al*. (9), Schulze *et al*. (15) and Meijer *et al*. (28), we believe that for the measurements repeatability of this type of radiologic studies, a single and experienced examiner is better in order to ensure that the errors produced by the observer will be consistent at least.

As we have shown in our previous study (10) there tends to be a disagreement when more than one examiner participate in the study. Though the use of a single calibrated observer allows some consistency, it also represents a possible drawback as it always includes the possibility for methodological bias. Another possible drawback to this study is the fact that no data was gathered regarding mandibular incisive canal and that the patients were not randomly selected, which will make the replication of the study difficult.

We believe that identification of the anatomic structures depends on the degree of cortication of the mental canal and image resolution, coinciding with Benavides *et al*. (5) who state that quality of the image is more important than the clinician´s diagnostic ability and thoroughness.

The information from this study may be used to provide recommendations to clinicians without access to a 3D scan of the interforaminal region when planning implant placement in this area.

## Conclusions

The identification and the measurement of the anterior extension of the mental nerve are essential for planning surgical procedures in the interforaminal region to prevent injuries to the nerve and its associated complications.

In the past, clinicians have only relied on 2-D imaging techniques for most of the surgeries in the anterior mandible despite the fact that the mental loop cannot be properly assessed in all cases.

Even though we have not found statistically significant differences between CBCT and PR with respect to the identification and length of the ML, we consider that a CBCT should be recommended when planning implant placement in anterior mandible in order to maximize the use of the available space from the prosthetic point of view and to minimize the risk of injury in case of the safety-margin distance being too little.

It is necessary to increase the number of patients in further studies in order to make a strong recommendation with regards to safety margins.
